# Energetic Selection of Topology in Ferredoxins

**DOI:** 10.1371/journal.pcbi.1002463

**Published:** 2012-04-05

**Authors:** J. Dongun Kim, Agustina Rodriguez-Granillo, David A. Case, Vikas Nanda, Paul G. Falkowski

**Affiliations:** 1 Department of Chemistry and Chemical Biology, Rutgers University, Piscataway, New Jersey, United States of America; 2 Environmental Biophysics and Molecular Ecology Program, Institute of Marine and Coastal Sciences, Rutgers University, New Brunswick, New Jersey, United States of America; 3 Department of Biochemistry, Robert Wood Johnson Medical School, University of Medicine and Dentistry of New Jersey, Piscataway, New Jersey, United States of America; 4 BioMaPS Institute for Quantitative Biology, Rutgers University, Piscataway, New Jersey, United States of America; 5 Department of Earth and Planetary Sciences, Rutgers University, New Brunswick, New Jersey, United States of America; Max-Planck-Institut für Entwicklungsbiologie, Germany

## Abstract

Models of early protein evolution posit the existence of short peptides that bound metals and ions and served as transporters, membranes or catalysts. The Cys-X-X-Cys-X-X-Cys heptapeptide located within bacterial ferredoxins, enclosing an Fe_4_S_4_ metal center, is an attractive candidate for such an early peptide. Ferredoxins are ancient proteins and the simple α+β fold is found alone or as a domain in larger proteins throughout all three kingdoms of life. Previous analyses of the heptapeptide conformation in experimentally determined ferredoxin structures revealed a pervasive right-handed topology, despite the fact that the Fe_4_S_4_ cluster is achiral. Conformational enumeration of a model CGGCGGC heptapeptide bound to a cubane iron-sulfur cluster indicates both left-handed and right-handed folds could exist and have comparable stabilities. However, only the natural ferredoxin topology provides a significant network of backbone-to-cluster hydrogen bonds that would stabilize the metal-peptide complex. The optimal peptide configuration (alternating α_L_,α_R_) is that of an α-sheet, providing an additional mechanism where oligomerization could stabilize the peptide and facilitate iron-sulfur cluster binding.

## Author Summary

The ferredoxin fold is one of the oldest structures capable of catalyzing electron transfer reactions. In nature, only a right-handed topology exists in the ferredoxin fold. To understand how a specific fold-handedness was selected, we analyzed the structural motif using the tools of *de novo* protein design, searching in an unbiased fashion for backbone geometries that can favorably interact with the tetrahedral iron-sulfur cluster. *In silico*, we found both left-handed and right-handed folds can be formed, however the right-handed folds provide up to six hydrogen bonds that can stabilize the reduced iron-sulfur cluster, whereas left-handed folds at most form three hydrogen bonds. The difference in electrostatic conformational energy may have influenced selection of topology early in the evolution of iron-sulfur cluster containing proteins. This observation led us to establish a fundamental protein design principle that only right-handed peptide folds can properly interact while maintain redox function. Our results provide guidance in the creation of artificial proteins capable of carrying out redox reactions.

## Introduction

Metals in proteins play important roles in stabilizing structure, promoting electron transfer and performing catalysis. Whole-genome analyses of phylogenetically diverse microorganisms suggest the earliest proteins incorporated metals and that metal usage over biological history evolved to match the availability of inorganic components in the environment [1], [2], [3]. The mechanisms by which the ligand environment modulates metal affinity and specificity are of significant interest in the study of metalloprotein evolution, function and design. Geometric requirements of metal coordination are predicted to impose specific constraints on the structure and topology of a bound polypeptide chain. In this study, we computationally model the accessible conformations of a ferredoxin-like peptide bound to an Fe_4_S_4_ cubane cluster in order to better understand how a putative early metalloprotein may have evolved.

It has been proposed that a set of core genes encode proteins that carry out key redox reactions essential for promoting life and driving biogeochemical cycles [4]. These proteins would be among the earliest to emerge in the ancient oceans. Identifying members of this set of core genes is an important step in understanding the evolution of microbial metabolism and emergent biogeochemical cycles. A number of features of ferredoxins make them an attractive as key players in the evolution of redox active proteins. Sequence analysis suggests that ferredoxins evolved very early in the origins of biological catalysis of redox reactions [5], [6]. All ferredoxins have a simple, conserved fold that binds two Fe_4_S_4_ clusters and is composed of fifty to sixty amino acids. Sequence and structural symmetry suggest it may have evolved from a gene duplication event of a thirty amino acid sequence, each capable of binding one iron-sulfur cluster [7], [8], [9], [10]. An early study of the ferredoxin sequence by Eck and Dayhoff in 1961 revealed even shorter repeats of four amino-acids [5], suggesting a prebiotic “protoferredoxin” was potentially composed of a primeval subset of the twenty amino acids [11], [12]. Midpoint potentials (−700 to −300 mV) of ferredoxins are lower than most other proteins, consistent with the mildly reducing early oceans [13], [14].

It has been speculated that the iron-sulfur cluster utilized in many redox proteins [15] may be an evolutionary relic of prebiotic chemistry catalyzed by mineral surfaces. Mineral surfaces can effectively adsorb and concentrate organic molecules and catalyze various chemical reactions implicated in the origin of non-equilibrium redox reactions. Chiral mineral surfaces can selectively interact with chiral amino acids, and thus have been extensively studied as a potential origin of life on Earth [16]. Iron-sulfur mineral surfaces especially have gained much attention in the context of deep-sea iron-sulfur rich hydrothermal vents where the earliest biologically relevant redox reactions are postulated to have occurred [17], [18].

**Figure 1:**
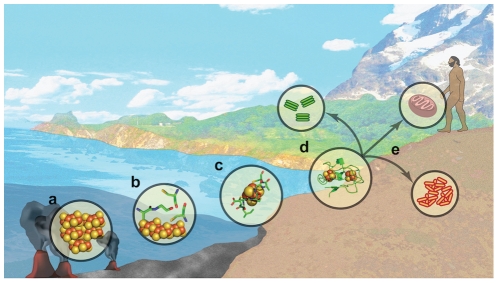
Hypothesized progression of iron-sulfur clusters from hydrothermal vents to life.

Assuming ferredoxin is one of the select core genes that originated from a mineral surface catalyst - what might intermediates in this progression from mineral to protein look like? (Figure 1): (A) Iron-sulfur minerals such as pyrite and mackinawite can spontaneously catalyze carbon fixation to generate essential organic molecules for life [19], [20], [21], [22], (B) The regular mineral concentrates amino acids [23], permitting new chemistry or enhancing existing reactions. (C) Condensation of small polypeptides occurs at the water-mineral interface [24]. These polypeptides could have sequences similar to Dayhoff's proposed tetrapeptides [25] and would be capable of stabilizing specific oxidation states of bound iron-sulfur fragments. (D) Small polypeptides are used as components of ferredoxin-like proteins. This is the transition from prebiotic chemistry to life and could occur within the context of models for such a transition such as an RNA-world where peptides are co-opted by small RNA hairpins [26]. (E) Ferredoxin is retained in all kingdoms and becomes a domain of larger proteins that include many of the core redox genes of life. Although each of these stages is poorly understood and arguably controversial, this conceptual framework allows the design of specific simulations and experiments to explore the feasibility of ferredoxin evolution from a mineral precursor.

The structural properties of a putative proto-ferredoxin peptide in Stage C have implications beyond origins of life models to metalloprotein design. Although several iron-sulfur binding sites have been designed into existing proteins [27], [28] and *de novo* folds [29], [30], [31], very few have shown any significant stability to cycles of oxidation-reduction, diminishing their utility in catalysis or bioenergy applications [32], [33]. By elucidating the geometric and energetic constraints on a polypeptide bound to an iron-sulfur cluster, one can potentially understand the physical rules governing biological redox reactions and the designing novel protein structures.

**Figure 2:**
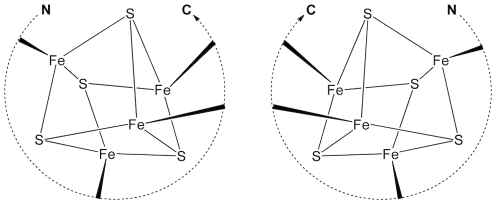
Two topological states of peptide-Fe_4_S_4_ cluster.

In the ferredoxin fold, iron-sulfur cluster has a quasi-tetrahedral structure with four coordination sites, which are most commonly occupied by four cysteine thiolates. The iron-sulfur cluster itself is achiral and the protein topology is mainly dependent on how the cysteine groups from a peptide chain are linked with four iron atoms in the cluster [34]. Topologically, two different modes of protein-cluster interactions, right-handed or left-handed, are possible (Figure 2). These two topological states cannot be superimposed onto each other by bending or stretching the representative molecular graphs [34]. Previous studies analyzing iron-sulfur proteins in the Protein Data Bank (PDB) reported that all redox active proteins had a right-handed fold; although left-handed configurations existed for redox inactive proteins [35].

Herein, we present the work that elucidates why a right-handed heptapeptide topology may have evolved in the context of metal-protein energetics.

## Results/Discussion

### Definition of handedness in protein folds

**Figure 3:**
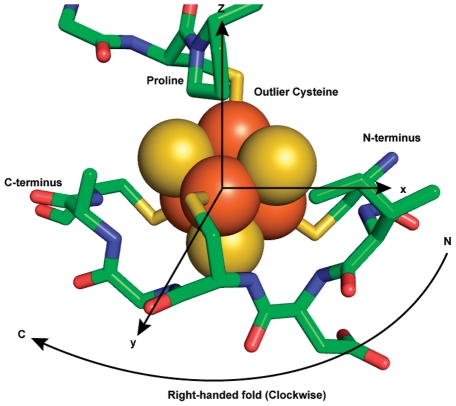
Fold topology in a ferredoxin fold. Right/Left fold configuration can be defined with an outlier, by orienting the outlier cysteine along the z-axis and iron-sulfur cluster being at the origin. A ferredoxin fold, with a conserved sequence CxxCxxC with an outlier cysteine, can create either right or left topological configuration. Right-handed fold is shown.

**Figure 4:**
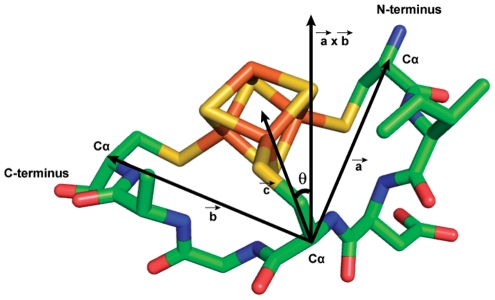
Topology angle in a ferredoxin fold for database analysis. An arbitrary plane was defined with three cysteine carbon alpha coordinates. Three dimensional vector calculations were done to determine the topology angle of the protein fold.

The achiral iron-sulfur (Fe_4_S_4_) cluster has a D_2d_ point group symmetry and is generally bonded to four cysteine thiolate groups [36], [37]. Three of the coordination sites are occupied by cysteine thiolates from a conserved heptapeptide sequence motif (CXXCXXC) and the remaining fourth coordination site is occupied by an outlier cysteine, which is most frequently followed by a proline (CP) [38]. This particular binding motif accounts for approximately 25% (36 out of 137) of iron-sulfur binding motifs from 104 crystal structures available from PDB (Table S1). Among the CXXCXXC motifs, about 85% (31 out of 36) have a ferredoxin fold and approximately 15% have globin-like folds and others as defined by Structural Classification of Proteins (SCOP) [39]. Topologically, the CXXCXXC heptapeptide motif can interact with an iron-sulfur cluster in two different ways, right-handed or left-handed (Figure 2). For the discussion of these topological states, we quantitatively describe the handedness of the folding using a “topology angle”, θ aligning the outlier cysteine on a z-axis of an internal coordinate frame (Figure 3). Once the outlier cysteine is specified, handedness in this study is defined relative to the N- to C-terminus chain direction, either proceeding clockwise (right-handed: 0°<θ<90°) or counterclockwise (left-handed: 90°<θ<180°) around the cluster (Figure 4). The outlier cysteine residue can be located before or after the CXXCXXC motif (CP…CXXCXXC or CXXCXXC…CP).

### Analysis of experimentally determined protein structures from PDB

**Figure 5:**
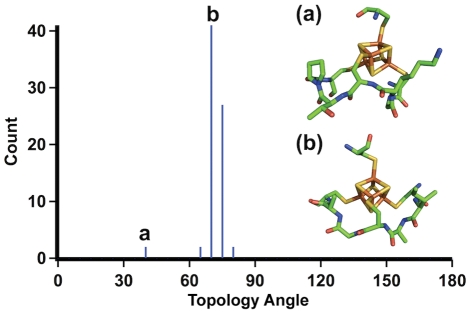
Topology of experimentally determined protein structures (Protein Data Bank). The absence of peaks between 90 to 180 degrees suggests that the left-handed fold conformation does not exist in the known structures archived in the PDB.

Since the initial analysis on protein structure database [35], the number of solved protein structures has increased at an exponential rate. A non-redundant subset (30% sequence similarity filter) of the PDB was searched for structures with an iron-sulfur (Fe_4_S_4_) cluster coordinated by a CXXCXXC sequence. The topology angle, θ, was calculated from the PDB coordinates (Figure 4). A histogram of the topology angles reveals that only right-handed folds are involved in an iron-sulfur cluster binding (Figure 5). The CXXCXXC motif always has a topology angle around 75°.

### Computational simulation with protCAD & AMBER

**Figure 6:**
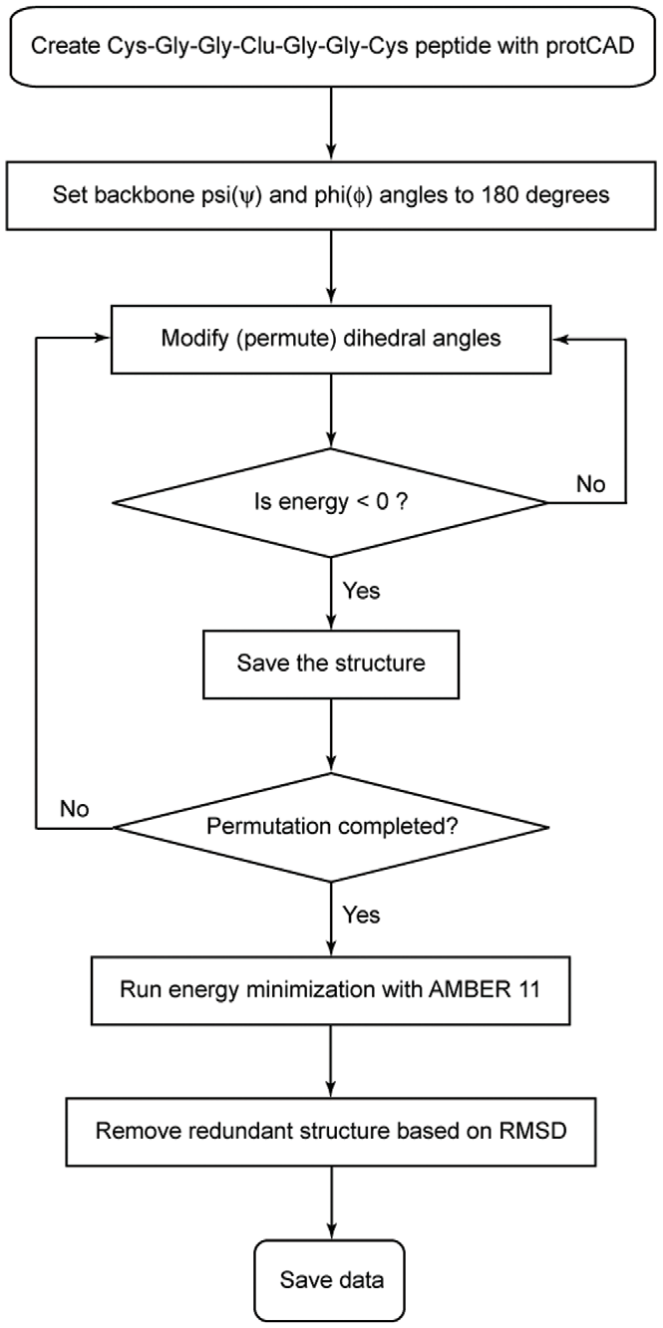
Protein ensemble generated by modifying psi, phi and chi dihedral angles. For a model heptapeptide-cluster complex, CGGCGGC fused to an iron-sulfur cluster, there are total 6 ψ angles, 6 Φ angles, 3 χ_1_ angles, and one each for χ_2_ and χ_3_ angles. The permutations are carried out by 60 degrees step size for Φ and ψ and 120 degrees step size for χ angles.

**Figure 7:**
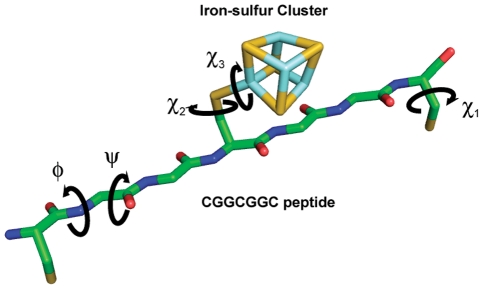
Cys-Gly-Gly-Clu-Gly-Gly-Cys peptide created with protCAD. All possible structures are explored by permuting 17 rotatable dihedral angles of the peptide from −180 to 180 with a step size of 60 degrees.

Left-handed configurations of CXXCXXC were not observed, leading us to examine whether such configurations were energetically plausible. An ensemble of CGGCGGC polypeptide configurations was generated. Glycine was chosen for non-Cys positions due to its high backbone flexibility, ensuring the primary conformational constraints came from metal-peptide interactions. The protCAD software platform(protein Computer Assisted Design) [29], [40] was used to exhaustively enumerate all combinations of backbone and sidechain torsions in 60° intervals for Φ,ψ and 120° intervals for the cysteine χ_1_ rotamer (Figure 6 and Figure 7). Out of 5.8×10^10^ (3^3^×6^12^) configurations, 232 exhibited net-favorable van der Waals interactions (less than 0 kcal/mol), Fe_cluster_··· S_γ_ distances (<3 A) and C_β_-S_γ_···Fe_cluster_ angles (120° to 180°) that would permit binding to an iron-sulfur cluster. The protein structures were then minimized in AMBER to reduce strain from distortions caused by discrete conformation sampling [41].

**Figure 8:**
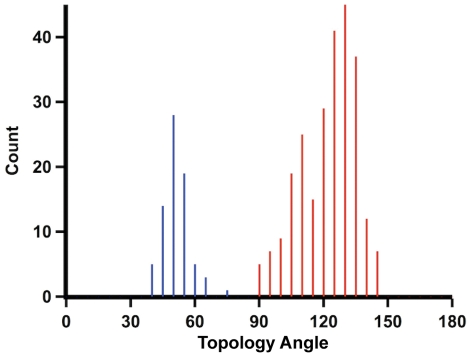
Topology angles of entactic structural states. Cys-Gly-Gly-Clu-Gly-Gly-Cys hepeptide model has 232 structural entactic states, either right-handed (blue, 75 out of 232) or left-handed (red, 157 out of 232). Despite the inexistence of left-handed topological state in nature, model peptide suggests that left-handed structure can also properly interact with an iron-sulfur cluster.

**Figure 9:**
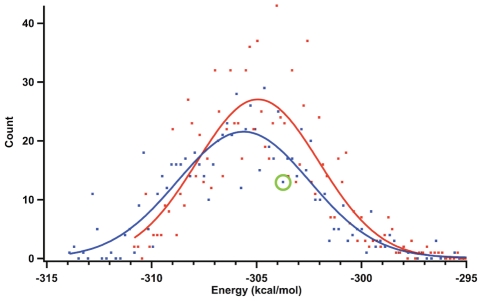
The energy distributions of right (blue) and left-handed (red) structures. The gaussian fits are very similar, which suggests that the natural selection was not influenced by the energetic stability alone. The energy corresponding to the ensemble that has the lowest RMSD to the experimentally determined ferredoxin structure (PDB: 2FDN)- green circle.

Topology angles of the computationally generated dataset clustered into two distinct populations - right and left-handed folds - suggesting the CGGCGGC heptapeptide could bind to the iron-sulfur cluster with either topology (Figure 8). In fact, the simulation identified *more* left-handed structures (67%) than right-handed structures (32%), indicating left-handed topologies were entropically favorable. Conducting the same simulation on CAACAAC resulted in 54% left-handed and 46% right-handed structures, suggesting that the steric hindrance of amino acid side chains itself is not sufficient to discriminate the handedness of the topological state. A histogram of the energy distributions for left and right-handed topologies show no significant difference (Figure 9), indicating intrinsic stability of the fold alone is unlikely to account for evolution of a unique topology.

### Hydrogen bonds in iron-sulfur proteins

The reduced state of the iron-sulfur cluster can be stabilized by hydrogen bonds contributed by nearby backbone amides [42]. The number of hydrogen bonds around the iron-sulfur cluster is also related to the solvent accessibility to the cluster, thereby tuning the midpoint potential [43], [44]. A typical ferredoxin fold exhibits six such interactions with backbone amides directing the proton toward the cluster. Hydrogen bond formation is at the expense of unfavorable backbone dihedral angles, particularly the positive *Φ* values at X_2_ and X_3_ positions (Table S2).

**Figure 10:**
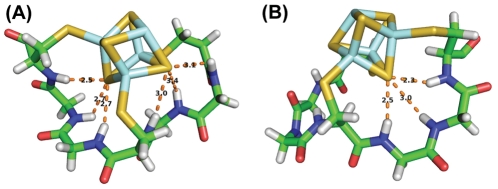
Computationally generated entactic states of the model heptapeptide with optimal peptide-cluster interaction energies. (A) Right-handed fold can form six hydrogen bonds, whereas (B) left-handed fold can only contribute three hydrogen bonds.

For the analysis of the hydrogen bonding environment of computationally generated structures, interactions were counted based on discrete distance and angular cutoffs: a hydrogen-sulfur distance less than 3.5 Å and N-H···S angles between 120 to 180° [45]. The number of hydrogen bonds between nitrogen and sulfur were counted based on cutoffs: 3.8 Å and 110 to 180°. Right-handed folds could accommodate six hydrogen bonds, but a maximum of three hydrogen bonds were found in structures with left-handed folds (Figure 10).

### Energetics of peptide-cluster interactions

**Figure 11:**
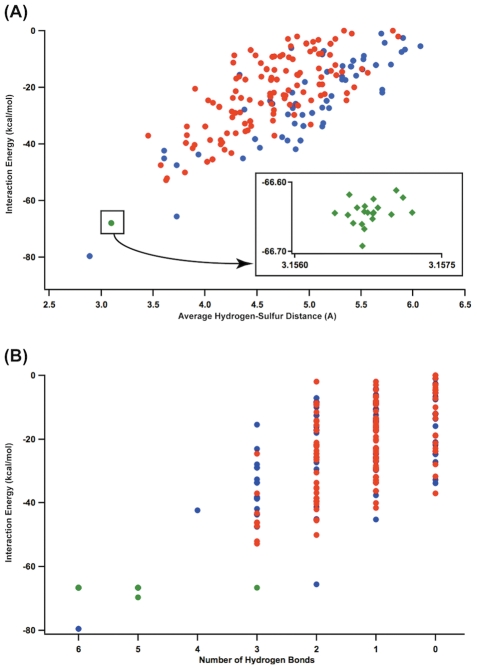
Hydrogen bonding environment of the 232 left- and right-handed heptapeptide-cluster conformations. (A) Interaction energy vs. average H-S distance of left (red), right-handed (blue) complexes. Experimentally determined ferredoxin structures (green) and non-ferredoxin redox active proteins (purple) show nearly identical bond geometries and calculated interaction energies. (B). The same dataset presented as the number of hydrogen bonds versus interaction energy. Only one simulated peptide in the ensemble contributes six hydrogen bonds, corresponding to the best interaction energy. This is equivalent to the natural right-handed fold.

The electrostatic stabilization of a bound cluster by proximal backbone amides was estimated by comparing the total energies of charged versus uncharged clusters in the context of a coordinating peptide. The net contribution of hydrogen bonds can represented several ways: the average of pairwise distances between hydrogen and sulfur atoms (Figure 11A) and discrete number of hydrogen bonds plotted against the peptide-cluster interaction energies (Figure 11B). The interaction energy improves as the distances between sulfur atoms to hydrogen atoms are reduced. The result also indicates that the right-handed peptide-cluster interaction can have a stabilization effect up to −80 kcal/mol, whereas a left-handed fold can only achieve −50 kcal/mol. For comparison, we generated a CGGCGGC peptide using coordinates from experimental ferredoxin structures, including proteins with non-ferredoxin fold (Figure 11A inset, Supplementary data). The right-handed topology in natural ferredoxin and non-Fd proteins presents a network of stabilizing backbone amides that interact strongly with the Fe_4_S_4_ cluster. The result shows the best right-handed structure contributes more stabilizing hydrogen bonds than the best left-handed structure. Additionally, the inset to figure 11A reveals tightly clustered experimental results, all which cluster around the same right-handed configuration and present six hydrogen bonds, suggesting the right-handed heptapeptide topology is a unique entactic state.

### Conclusion

A microscopic analysis of the Fe_4_S_4_ binding region of ferredoxin provides some insights into the predicted features of an ancient, short proto-ferredoxin. The right-handed topology observed in redox-active iron-sulfur proteins is not dictated by the peptide chain. In fact, left-handed chain topologies are entropically favored and have slightly improved stabilities in the absence of the cluster. Only when considering electrostatic interactions with the cofactor is the natural right-handed topology the optimal solution. Thus short CxxCxxC peptides alone are unlikely to serve as early redox active species without additional external stabilizing interactions. These may have taken the form of longer sequences with super-secondary structure such as those in designed peptide maquettes [38], [46]. It is interesting to note that the model conformation with the best peptide-cluster interaction energy and the ferredoxin-like conformations are both an α-sheet, characterized by residues in alternating α_L_ and α_R_ conformations. This motif was first described by Pauling and Corey as the ‘pleated sheet’ [47]. α-sheets are thought to be intermediates in a number of protein aggregation disorders [48], [49]. The conformation is also implicated in early peptides due to their anion binding properties [50]. It is possible that stabilization of α-sheets provides the entactic state required for favorable cluster binding. The identification of a specific iron-sulfur binding topology may point the way to a mechanism by which the first core metalloproteins evolved.

## Materials and Methods

### Topology angle

To have a quantitative measure for the fold-handedness, an arbitrary plane was defined with two vectors, which were defined by C_α_ coordinates from three cysteine residues. The topology angle, a quantitative measure of fold-handedness, was then defined as the angle between a normal vector of the arbitrary plane and a vector from the middle cysteine C_α_ to the cluster. By definition, the quantitative measurement of fold-handedness (topology angle) can take any numeric value from 0° to 180°.

### protCAD

Iron-sulfur cluster coordinates were extracted from the PDB file, 2FDN. We created a hybrid artificial amino acid residue (Clu) by linking an iron-sulfur cluster to a cysteine residue. The artificial amino acid was added to the amino acid library of protCAD. Initially a peptide ensemble (Cys-Gly-Gly-Cys-Gly-Gly-Cys) was created and subsequently the central Cys was substituted to Clu. For a given ensemble, there are six *Φ* (C′-N-C_α_-C′), six *ψ* (N-C_α_-C′-N). For each cysteine residue, there three *χ_1_* (N-C_α_-C_β_-S_γ_) dihedral angles. For the central iron-sulfur cluster fused cysteine residue, there are additional dihedral angles, which are χ_2_ (C_α_-C_β_-S_γ_-Fe_Clu_) and χ_3_ (C_β_-S_γ_-Fe_Clu_-S_Clu_). All phi and psi dihedral angles were increased by a step size of 60° and all chi dihedral angles were set at −180°, −60°, or 60°. The entire protein structural space was searched by the permutations of seventeen dihedral angles. Plausible protein structures were then determined by geometric parameters, such as a distance from S_γ_ to Fe_Clu_ with a cutoff (<3.0A). Energy parameters calculated based on a Lennard-Jones equation [45] was also used to detect feasible structures (total energy<0 kcal/mol).

### AMBER 11

The structures obtained from the ProtCAD simulations were subjected to energy minimization calculations using Amber 11 [51], with a generalized Born solvent model [52], [53]. Protein atoms were described with the parm99SB [54], [55], [56] force field parameterization. The atomic charges were modified so that an oxidized Fe_4_S_4_^+2^ cluster bound to 3 Cys had a net charge of −1, yielding the following charges: qFe = 0.6518 e, qS (cluster) = −0.5552 e, qSG (cysteine) = −0.6042 e. The maximum number of minimization cycles was set to 10^5^, and the structures were considered minimized when the root-mean-square of the Cartesian elements of the gradient was less than 10^−4^ kcal/mol-Å. To compare the degree of electrostatic stabilization of the cluster in the different peptide models, the charge of the S atoms of the Fe_4_S_4_ cluster was set to zero, and a single point energy calculation was performed. A number of structures converged to an identical structure after the energy minimization process. The redundant structures were then removed by MMTSB (Multiscale Modeling Tools in Structural Biology) k-clustering algorithm [57].

## Supporting Information

Table S1 List of structures collected from Protein Data Bank(PDB). Structures containing a CXXCXXC binding motif with 30% sequence similarity were collected. The most common iron-sulfur cluster binding motif is CXXCXXC with two types of outlier positions: Type A: (CXXCXXC….C) Type B: (C….CXXCXXC).
(DOC)

Table S2 Alternating α_L_,α_R_ secondary structure, also known as alpha-sheet, characterized by positive phi dihedral angles in C-X_1_-X_2_-C-X_3_-X_4_-C motif (Protein structures from PDB). Alpha-left (α_L_) friendly amino acids (e.g. Asp, Asn, His, Lys) (1) are color coded with pale blue and residues that are unlikely to accommodate positive phi dihedral angle are noted with orange. Glycine and cysteine are colored pale green and yellow, respectively.
(DOC)
