# Supplementary material for: Energetic Selection of Topology in Ferredoxins
Source: PLoS Comput Biol. 2012 Apr 5;8(4):e1002463. doi: 10.1371/journal.pcbi.1002463 (PMC3320576; doi:10.1371/journal.pcbi.1002463)
Supplement: Table S1 — List of structures collected from Protein Data Bank(PDB). Structures containing a CXXCXXC binding motif with 30% sequence similarity were collected. The most common iron-sulfur cluster binding motif is CXXCXXC with two types of outlier positions: Type A: (CXXCXXC….C) Type B: (C….CXXCXXC). (DOC) [file pcbi.1002463.s001.doc]

**Supplementary Data**

| PDB ID | Motif Type | Fold |
| --- | --- | --- |
| 1GTE | A | Fd |
| 1GTE | B | Fd |
| 1GX7 | A | Fd |
| 1GX7 | B | Fd |
| 1H98 | B | Fd |
| 1HFE | A | Fd |
| 1HFE | B | Fd |
| 1IQZ | A | Fd |
| 1JNR | B | Fd |
| 1KQF | A | Fd |
| 1KQF | B | Fd |
| 1O94 | A | Others |
| 1VLF | A | Fd |
| 2C42 | A | Fd |
| 2C42 | B | Fd |
| 2FDN | A | Fd |
| 2FDN | B | Fd |
| 2GMH | B | Fd |
| 2H88 | A | Others |
| 2IVF | A | Fd |
| 2VKR | A | Fd |
| 2VPZ | A | Fd |
| 2VPZ | B | Fd |
| 2Z8Q | A | Fd |
| 3BK7 | B | Fd |
| 3C8Y | A | Fd |
| 3C8Y | B | Fd |
| 3CF4 | A | Others |
| 3CF4 | B | Others |
| 3EUN | A | Fd |
| 3IAM | A | Fd & Others |
| 3IAM | B | Fd |
| 3MM5 | B | Fd |
| 7FD1 | B | Fd |

***Table S1.*** *List of structures collected from Protein Data Bank(PDB). Structures containing a CXXCXXC binding motif with 30% sequence similarity were collected. The most common iron-sulfur cluster binding motif is CXXCXXC with two types of outlier positions: Type A: (CXXCXXC….C) Type B: (C….CXXCXXC).*
