# Supplementary material for: Energetic Selection of Topology in Ferredoxins
Source: PLoS Comput Biol. 2012 Apr 5;8(4):e1002463. doi: 10.1371/journal.pcbi.1002463 (PMC3320576; doi:10.1371/journal.pcbi.1002463)
Supplement: Table S2 — Alternating αL,αR secondary structure, also known as alpha-sheet, characterized by positive phi dihedral angles in C-X1-X2-C-X3-X4-C motif (Protein structures from PDB). Alpha-left (αL) friendly amino acids (e.g. Asp, Asn, His, Lys) (1) are color coded with pale blue and residues that are unlikely to accommodate positive phi dihedral angle are noted with orange. Glycine and cysteine are colored pale green and yellow, respectively. (DOC) [file pcbi.1002463.s002.doc]

| **PDB ID** | **Phi** | | | | | | | **Source Organism** |
| --- | --- | --- | --- | --- | --- | --- | --- | --- |
|  | **+** | **+** | **-** | **+** | **-** | **+** | **+** |  |
|  | **C** | **X_1_** | **X_2_** | **C** | **X_3_** | **X_4_** | **C** |  |
| 1CLF | C | V | S | C | G | A | C | *C. pasteurianum* |
| 1CLF | C | I | D | C | G | N | C | *C. pasteurianum* |
| 1DUR | C | I | D | C | G | S | C | *P. asaccharolyticus* |
| 1DUR | C | I | A | C | G | A | C | *P. asaccharolyticus* |
| 1DWL | C | I | G | C | E | S | C | [*D. norvegicum*](http://www.pdb.org/pdb/search/smartSubquery.do?smartSearchSubtype=TreeEntityQuery&t=1&n=52561) |
| 1FXR | C | I | A | C | E | S | C | *D. africanus* |
| 1H98 | C | I | D | C | G | A | C | *T. thermophilus* |
| 1IQZ | C | I | A | C | G | A | C | *B. thermoproteolyticus* |
| 1VJW | C | I | G | C | G | V | C | *T. maritime* |
| 2C42 | C | I | Q | C | N | Q | C | *D. africanus* |
| 2FDN | C | I | D | C | G | A | C | *C. acidi-urici* |
| 2FDN | C | I | S | C | G | A | C | *C. acidi-urici* |
| 2FGO | C | I | N | C | D | V | C | *P. aeruginosa* |
| 2VKR | C | I | F | C | M | A | C | *A. ambivalens* |
| 1VJW | C | I | G | C | G | V | C | *P. furiosus* |

***Table S2.*** *Alternating α_L_,α_R_ secondary structure, also known as alpha-sheet, characterized by positive phi dihedral angles in C-X_1_-X_2_-C-X_3_-X_4_-C motif (Protein structures from PDB). Alpha-left (α_L_) friendly amino acids (e.g. Asp, Asn, His, Lys) (1) are color coded with pale blue and residues that are unlikely to accommodate positive phi dihedral angle are noted with orange. Glycine and cysteine are colored pale green and yellow, respectively.*

**Reference Cited**

1. Annavarapu S., Nanda V. Mirrors in the PDB: left-handed alpha-turns guide design with D-amino acids. BMC Struct Biol. 2009;9(61)
